# Perioperative temperature management and coagulation: effects of mild hypothermia in a prospective study

**DOI:** 10.3389/fmed.2025.1536782

**Published:** 2025-06-16

**Authors:** Alena Trčková, Tereza Bönischová, Hana Zelinková, Petr Štourač

**Affiliations:** ^1^Department of Pediatric Anesthesiology and Intensive Care Medicine, University Hospital Brno and Faculty of Medicine, Masaryk University, Brno, Czechia; ^2^Department of Simulation Medicine, Faculty of Medicine, Masaryk University, Brno, Czechia; ^3^Institute of Biostatistics and Analyses, Faculty of Medicine, Masaryk University, Brno, Czechia

**Keywords:** body temperature, coagulopathy, coagulation test, ROTEM, perioperative, children, pediatric anesthesia, temperature management

## Abstract

**Background:**

Perioperative hypothermia is a common complication of general and regional anesthesia in children and is a known risk factor for the development of coagulation disorders. The primary aim of the study was to assess the occurrence of coagulopathy in hypothermic pediatric patients (0–18 years) undergoing arthroscopic surgery and open abdominal surgery. The secondary objective was to identify potential risk factors for the development of both hypothermia and coagulopathy.

**Methods:**

A prospective cohort study was conducted, forming the second part of our study “Perioperative Management of Temperature in Children and the Influence of Hypothermia on Blood Clotting in Children” (Peritemp). We observed the incidence of body temperatures below normal values—specifically, below 36.5°C and 36°C—as well as the incidence of pathological values in thromboelastometry (ROTEM) (EXTEM and FIBTEM) and standard coagulation tests, including activated partial thromboplastin time (aPTT) and prothrombin time (PT).

**Results:**

A total of 102 patients (55 female and 47 male patients) were enrolled from 22nd January 2018 to 27th August 2021 at the Department of Pediatric Anesthesiology and Intensive Medicine, University Hospital Brno. An incidence of body temperature below 36.5°C was observed in 86 cases, and temperatures below 36.0°C were observed in 43 cases. The incidence of abnormalities in the individual parameters of the coagulation tests ranged from 5.9 to 32.4%. The ROTEM results were abnormal in 18.7% of the patients, while the standard coagulation test showed abnormalities in 15.9% of the cases. In the statistical comparison between the first and second coagulation test results, only the prothrombin time ratio (PT-R) showed a statistically significant difference. Low operating room (OR) temperature and patient age emerged as significant risk factors for the incidence of hypothermia. In addition, older age was associated with an increased likelihood of body temperature falling below 36.5°C and 36°C.

**Conclusion:**

Our study confirmed that mild hypothermia (core temperature below 36.0°C) is common during pediatric surgeries, but it does not appear to result in clinically significant coagulation disorders requiring intervention. Despite the incidence of coagulation abnormalities, the absence of significant changes in coagulation parameters, outside of the PT-R, suggests that mild hypothermia may be well tolerated by the coagulation system in pediatric patients. Our study confirmed the previously established association between variability in operating room temperature and intraoperative hypothermia. Future research should focus on larger, more diverse pediatric populations to validate these findings and optimize perioperative temperature management strategies.

**Clinical trial registration:**

ClinicalTrials.gov identifier: NCT03273894.

## 1 Introduction

The body temperature of patients undergoing surgery under general or regional anesthesia is one of the monitored and influenced vital functions. The core temperature ranges between 36.5°C and 37.5°C (1) in a non-anesthetized state. When the core temperature falls outside this range, mechanisms are activated to either increase heat production (e.g., increased muscle tone, increased metabolism) or promote heat loss (e.g., vasodilatation, sweating). However, these thermoregulatory mechanisms are impaired or suppressed during general and regional anesthesia. Thermoregulatory deprivation, exposure to low operating room (OR) temperature, and insufficient heating cause hypothermia (2). Hypothermia (body temperature below 36.0°C) has been observed with an incidence rate of 45–85% in children (3–5) and has a higher prevalence in neonates. Hypothermia increases the risk of coagulation disorders, surgical site infections, prolonged drug metabolism, thermal discomfort (6), and higher morbidity and mortality (7, 8). In neonates and preterm infants, it can lead to pulmonary hypertension, right-to-left pulmonary shunting, and poor neurologic outcomes (6). Body temperature below 35°C leads to platelet dysfunction, while temperature below 33°C reduces the synthesis of clotting enzymes and plasminogen activator inhibitors (10, 11). Coagulation parameters serve as tools for monitoring the status of a patient’s hemostatic system and are crucial for guiding proper follow-up treatment. There are several methods available to evaluate various aspects of coagulation. Conventional laboratory tests used to evaluate coagulation include prothrombin time (PT), activated partial thromboplastin time (aPTT), fibrinogen levels, clotting factors, anticoagulant pathways, platelet count, and markers of late tertiary hemostasis (D-dimers, plasminogen activity). In addition, the prothrombin time ratio (PT-R) and activated partial thromboplastin time ratio (aPTT-R) are calculated by comparing the patient’s PT or aPTT values to reference values. Long reporting times (0.5–4 h) are a limitation during surgery when the patient’s coagulation status is rapidly changing. To address this, point-of-care coagulation testing has become an invaluable tool in the perioperative setting. Tests such as activated clotting time (ACT) for assessing the efficacy of heparin therapy, international normalized ratio (INR) for monitoring PT in patients on vitamin K antagonists, platelet function testing (PFA) for detecting vW factor pathology and evaluating aspirin therapy, D-dimer testing, viscoelastic real-time techniques such as thromboelastography (TEG) and thromboelastometry (ROTEM) are commonly used. These methods offer rapid results that facilitate prompt clinical decision-making. Rotational thromboelastometry (ROTEM, ROTEM®; TEM International, Munich, Germany) and thromboelastography (TEG, TEG®; Haemonetics, Braintree, MA) are viscoelastic methods used for bedside diagnosis of hemostatic status. During the tests, a blood sample of a defined volume is placed into a cuvette, and a blood clot forms between the cuvette and a pin. In ROTEM, the pin is rotated, while in TEG, the cuvette is rotated and the pin remains stable. The formation and retraction of the coagulum affect the deflection of the pin, which is measured optically in ROTEM or mechanically in TEG. These data are then converted into graphic and numerical results. The aim of this prospective cohort study was to determine the occurrence of coagulopathy in our hypothermic pediatric patients.

## 2 Materials and methods

A prospective monocentric study was conducted at the Department of Pediatric Anesthesia and Intensive Medicine, University Hospital Brno, and was approved by the Ethics Committee (Ethics Commission University Hospital Brno, approved 8th March 2017, commission chairman PharmDr. Šárka Kozáková, MBA). Written informed consent was obtained from all study participants or, when applicable, from their legal representatives. The trial was registered on www.clinicaltrials.gov (ClinicalTrials.gov Identifier: NCT03273894).

This prospective monocentric study, serving as the second part of the study named Perioperative Management of Temperature in Children and Influence of Hypothermia on Blood Clotting in Children (Peritemp), focused on temperature management and coagulation. In the first part of our study, we monitored the incidence of hypothermia in our patients. We identified patients undergoing surgery involving the opening of the body cavity and younger age as risk groups. We also identified the temperature of the operating room as a significant factor influencing the occurrence of postoperative hypothermia.

### 2.1 Setting and participants

The study included pediatric patients aged 0–18 years (+ 364 days) scheduled for elective surgical procedures lasting more than 30 min. Eligible patients were those undergoing either arthroscopic surgery (selected due to the lowest operating theatre temperature in our hospital) or open abdominal surgery (based on findings from the first part of the Peritemp study). All participants provided informed consent during the pre-admission anesthesia visit. The exclusion criteria included the following: surgical intervention lasting less than 30 min, lack of consent from the patient or their legal representative, preexisting blood clotting disorders, and the risk of potential harm due to non-indicated blood sampling (e.g., newborns with low, very low, or extremely low birth weight). Patients were enrolled from 22nd January 2018 to 27th August 2021 at the Department of Pediatric Anesthesiology and Intensive Medicine, University Hospital Brno.

A total of seven age groups were defined as follows: 0–3 months, 4–12 months, 13–24 months, 2–5 years, 6–10 years, 11–16 years, and 17–18 years. These groups were used to apply age-related reference ranges as defined in “Thromboelastometry (ROTEM®) in children: age-related reference ranges and correlations with standard coagulation tests” (14).

### 2.2 Variables and data sources

We monitored the occurrence of body temperatures below the normal range, using 36.5°C and 36°C as cutoff points for hypothermia. In the CRF, we recorded the patient’s demographic data, ASA status, department, type of operation, operation site (trunk or limbs), and whether the procedure was laparoscopic, arthroscopic, or involved opening the body cavity. Body temperature was recorded before the procedure by the department at the time of premedication administration (approximately 45 min before arrival in the operating room), using either a non-contact infrared thermometer (Thermoval duo scan, Hartman®; Thermoflash LX 26-Evolution, Visiomed®) or a digital thermometer (Thermovalkids, Hartman®). In the operating room, temperature was measured at the beginning of anesthesia and every 15 min thereafter using various methods (nasopharyngeal, esophageal, axillary, skin probe, non-contact infrared thermometer, bladder or rectal probe). Additional temperature measurements were taken at the end of the procedure before leaving the operating room and again before leaving the recovery room. Other monitored parameters included the temperature management tools used during the surgery—passive methods such as covering the patient with a cotton drape or an anti-decubitus blanket, and active methods such as a hyper-hypothermia water mattress system (Blanketrol®), warmed infusions, a warm air heating system (Bair Hugger®), and an infusion tube heater. Blood loss was subjectively evaluated by the surgeon in milliliters (mL). Both the surgeon and anesthesiologist provided a subjective assessment of the patient’s bleeding (high, normal, or low). In addition, the need for blood products, results of intraoperative laboratory tests (if collected), and manifestations of thermal discomfort—such as shivering or a feeling of cold in patients able to express these sensations verbally— were recorded.

Once venous access was ensured, the first blood samples were taken for standard coagulation laboratory tests (aPTT-R, PT-R) and ROTEM delta® bedside tests (FIBTEM and EXTEM). Second samples were collected at the end of surgery from an already inserted venous catheter, whenever possible, to minimize harm to the patient. If this was not feasible, re-puncturing was performed for blood collection. Ethical considerations, including obtaining patient consent for blood collection and ensuring minimal harm during the procedure, were followed in accordance with institutional guidelines and ethical standards. This procedure was detailed in the informed consent form signed by the patient or their legal representatives and approved by the ethics committee. The test tube for standard coagulation tests was sent to the laboratory, while ROTEM samples were sent to the nearby resuscitation department after prior telephone notification, with the device maintained at operating temperature. ROTEM data were collected for at least 60 min. Both of these procedures followed standard departmental protocols independent of the study. The timing of result availability corresponded to non-study-related examinations requested by the anesthesiologist during regular working hours. At the end of surgery, second blood samples were collected. The first tests performed after venous access was ensured were labeled as “TEST 1” (e.g., EXTEM 1 – CT), while the tests performed at the end of surgery were labeled as “TEST 2” (e.g., EXTEM 2 – CT). Values were evaluated according to age-related reference ranges defined by Oswald et al. (14) for the following parameters: EXTEM CT, EXTEM CFT, EXTEM *α*, EXTEM A10, EXTEM MCF, FIBTEM A10, and FIBTEM MCF. Adult reference values were used for the EXTEM A20 and FIBTEM A20 tests (marked with an asterisk in the table and graph). The aPTT-R and PT-R values were evaluated using age-specific reference ranges from our laboratory. If the patient’s body temperature was below 34°C, two sets of ROTEM tests were conducted: one at the patient’s actual body temperature and a second at the standard temperature of 37°C.

### 2.3 Bias

The choice of temperature measurement site, temperature management method, and type of anesthesia was at the discretion of the anesthesiologist. The patient’s bleeding was subjectively assessed by both the anesthesiologist and the surgeon as normal, higher than normal, or lower than normal.

### 2.4 Statistical methods

The data were processed using Microsoft Excel and subsequently analyzed statistically using IBM SPSS Statistics, version 13.5.0.17. Group size was calculated based on a significance level of 0.05.

Statistical analysis of coagulation parameters was performed using the Wilcoxon matched-pairs test. The methods used to examine interactions were Pearson’s chi-squared test, the Wilcoxon matched pairs test, binary logistic regression, and Spearman’s correlation coefficient. Statistical analysis of shivering was performed using the Mann–Whitney U test.

The article was prepared following the guidelines for observational studies outlined in the STROBE statement (RRID: SCR_018788).

## 3 Results

**Figure 1:**
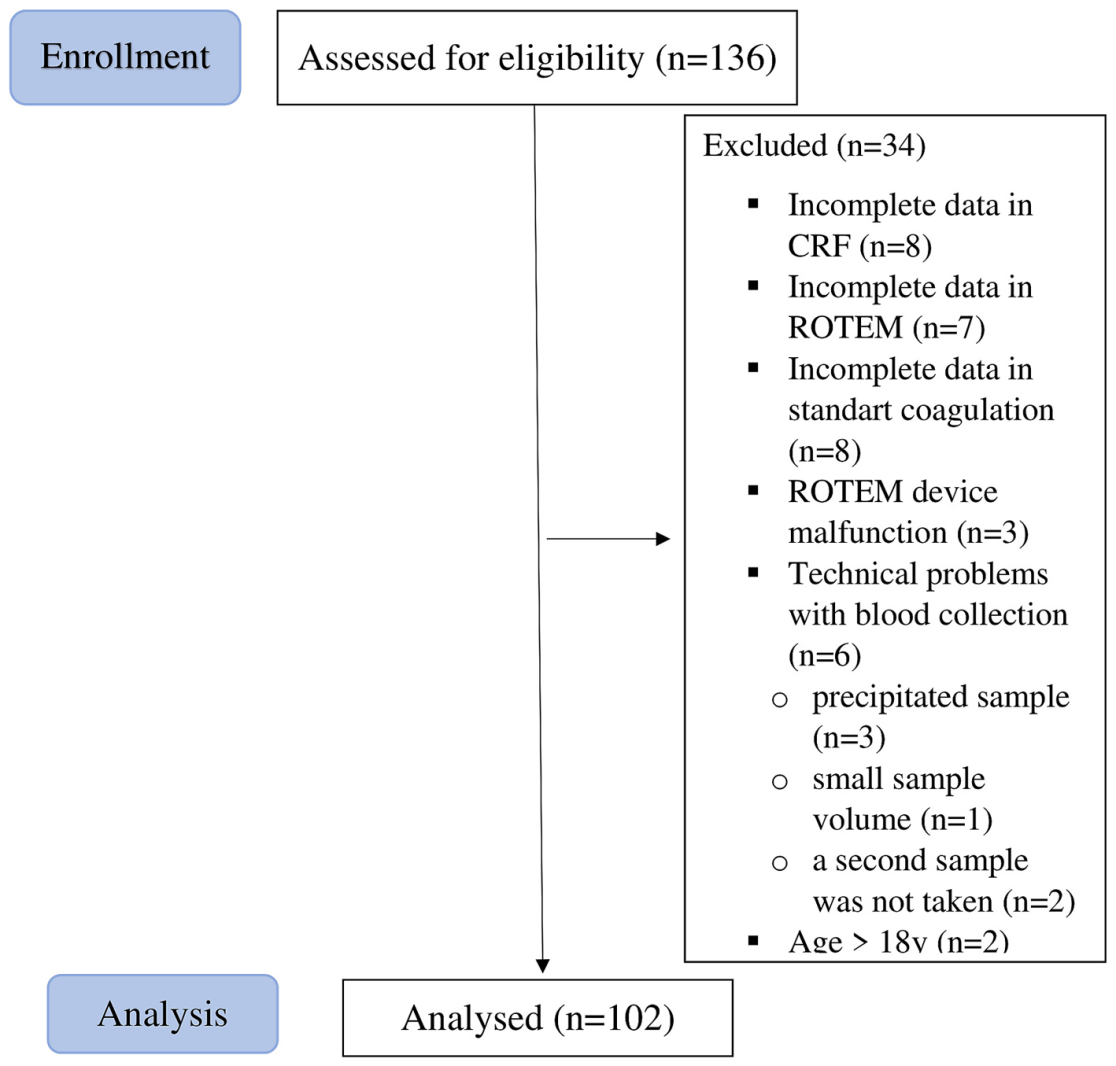
Flow chart diagram.

Of the 136 pediatric patients, 34 were excluded because of incomplete data in the CRF, insufficient or clotted blood samples, surgery duration shorter than 30 min, ROTEM device malfunction, or patient age exceeding 18 years on the day of surgery (postponed patients). See Figure 1 for the flow chart diagram.

### 3.1 Descriptive data

Characteristics of the participants (*n* = 102), including 55 female and 47 male individuals, are presented in Table 1 (Characteristics of the group).

**Table 1:** Characteristics of the group.

Variable	Descriptive Statistics	
	Median (min–max) °C	Interquartile range (lower quartile – upper quartile)
Weight (kg)	64 (11.6–110)	75 (21–54)
Height (cm)*	170 (85–190)	176 (12–164)
Age (years)	16 (1.5–18)	17 (3–14)
Surgery length (minutes)	80 (30–190)	115 (50–65)

^*^For height (cm), *n* = 80 patients. For all other parameters, *n* = 102 patients.

The patients were distributed across the predefined age groups as follows: 0–3 months (0 patients), 4–12 months (0 patients), 13–24 months (one patient), 2–5 years (two patients), 6–10 years (two patients), 11–16 years (55 patients), and 17–18 years (42 patients).

The majority of our patients were from the Department of Orthopedics, with only 3% from the Urology Department and 1% from the General Surgery Department. The average ASA status was 1.18. None of the patients had a known history of or ongoing hematological disease or coagulopathy, were taking medications affecting hemostasis or corticosteroids, or had thyroid malfunction or acute systemic infection. The most commonly used temperature measurement methods were the nasopharyngeal sensor (71%), esophageal sensor (9%), non-contact thermometer (8%), axillary sensor (7%), and skin sensor (5%).

### 3.2 Primary outcome—occurrence of hypothermia and coagulopathy

#### 3.2.1 Outcome data of body temperature

##### 3.2.1.1 Core temperature during anesthesia

An incidence of minimal body temperature below 36.5°C was observed in 86 cases (84.3%) and below 36.0°C in 43 cases (42.2%). Body temperature upon leaving the recovery room was below 36.5°C in 79 cases (77.4%) and below 36.0°C in 37 cases (36.3%). The incidence of body temperature below 36.5°C and below 36.0°C before leaving the recovery room was reduced to 8.1 and 14%, respectively.

Changes in body temperature over the course of general anesthesia are presented in Table 2 (Core temperature during anesthesia).

**Table 2:** Core temperature during anesthesia.

Time (min)	Descriptive statistics - core temperature during anesthesia	
	*N* (%)	Median (min–max), SD °C
0	101 (99%)	36.4 (35.1–37.5), 0.44
15	102 (100%)	36.4 (35.0–37.2), 0.41
30	102 (100%)	36.3 (35.1–37.1), 0.39
45	96 (94.1%)	36.2 (35.3–37.1), 0.40
60	88 (86.3%)	36.2 (35.2–37.2), 0.42
75	66 (64.7%)	36.2 (35.0–37.0), 0.47
90	38 (37.3%)	36.1 (34.9–37.3), 0.53
105	25 (24.5%)	35.9 (34.8–37.3), 0.53
120	20 (19.6%)	35.8 (34.8–37.3), 0.59
135	14 (13.7%)	36.0 (34.8–37.3), 0.61
150	9 (8.8%)	36.0 (35.0–37.2), 0.75
165	8 (7.8%)	36.1 (34.7–37.2), 0.83
180	3 (2.9%)	36.2 (35.0–37.0), 1.01
195	2 (2%)	36.6 (36.2–36.9), 0.49

**Figure 2:**
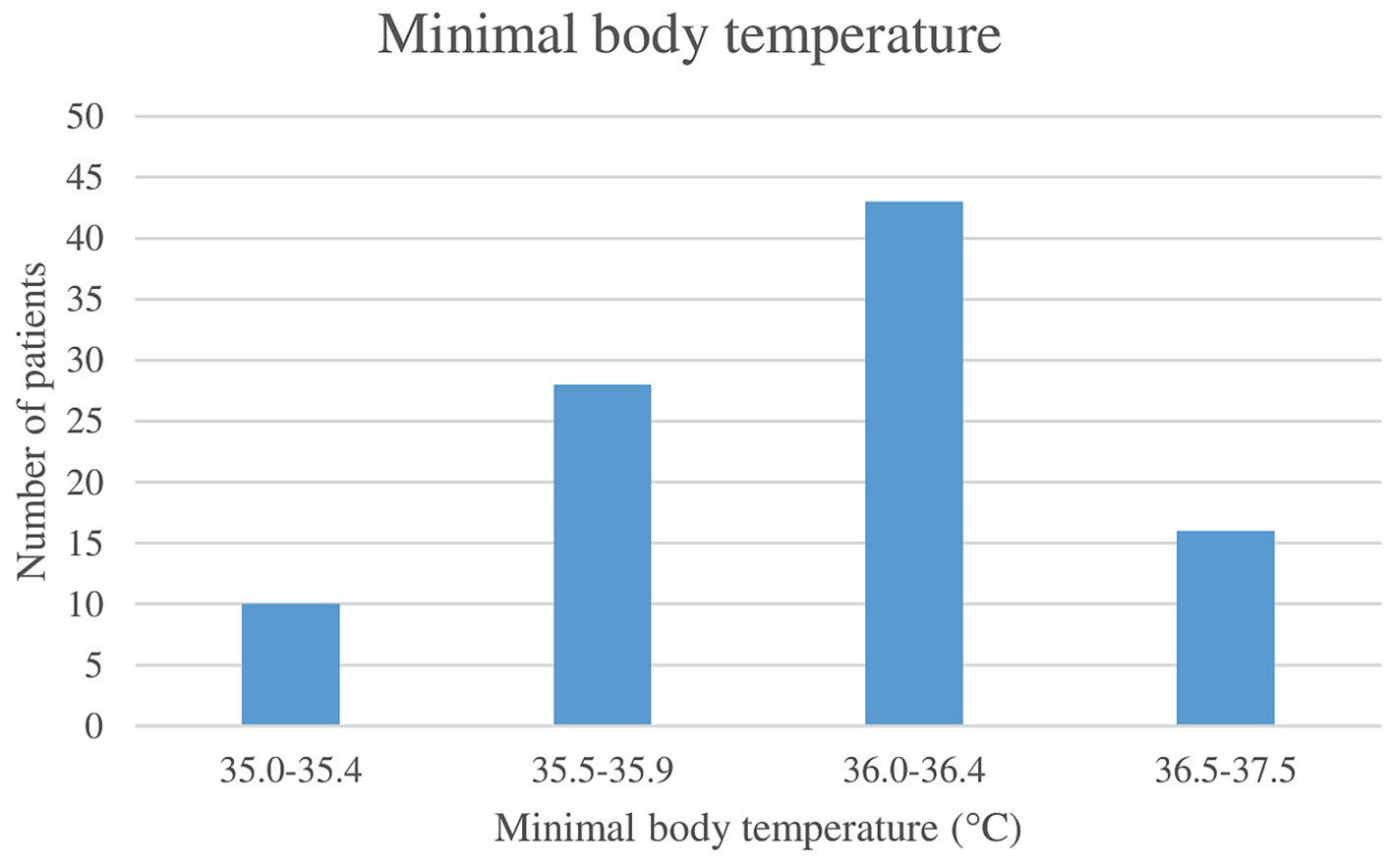
Minimal body temperature.

Table 3 (Descriptive statistics of selected measured quantities) presents the temperatures before the surgery, the minimal and maximal temperatures during the surgery and the first 60 min, the temperature of the operating theatre, and the duration of the surgery. Body temperature above 37.5°C during the surgery was measured in one patient. In Figure 2 (Minimal body temperature), the distribution of the minimum body temperatures recorded during the surgery is presented.

**Table 3:** Descriptive statistics of selected measured quantities.

Variable	Descriptive statistics		
	*N* (%)	Median (min–max)	Interquartile range (lower quartile – upper quartile)
Temperature before surgery (°C)	84 (82%)	36.6 (35.3–37.2)	0.2 (36.5–36.7)
Temperature of the operating room (°C)	102 (100%)	25.0 (22.8–28.0)	1 (24.6–25.6)
T min (°C)	102 (100%)	36.1 (34.7–37.1)	0.6 (35.7–36.3)
T max (°C)	102 (100%)	36.6 (35.6–37.5)	0.5 (36.3–36.8)
T during the first 60 min - min (°C)	102 (100%)	36.1 (35.0–37.1)	0.5 (35.9–36.4)
T during the first 60 min - max (°C)	102 (100%)	36.5 (35.6–37.5)	0.5 (36.3–36.8)
Surgery duration (min)	102 (100%)	80 (30–190)	50 (65–115)

##### 3.2.1.2 Discomfort caused by hypothermia

As a postoperative manifestation of discomfort caused by hypothermia, postoperative shivering was observed in 26.4% of the cases, and a subjective sensation of coldness was reported by 4.9% of the patients who were able to verbally express this feeling.

For further comparison, we included only patients undergoing knee arthroscopy, as this group had a sufficient sample size. Other surgical procedures were excluded as their occurrence was limited to only a few cases, preventing meaningful data representation. The incidence of shivering in this group was 27%. Only one patient had a postoperative temperature higher than 37°C and did not experience postoperative shivering. A statistically significant correlation was found between surgery duration and shivering incidence (*p* = 0.016). In addition, both the minimum body temperature and the temperature before leaving the operating room were significantly associated with shivering (*p* = 0.028 for both).

##### 3.2.1.3 Blood loss

As the patients were pediatric and of different ages, blood loss was adjusted for body weight and expressed in mL/kg. The average blood loss was 0.33 mL/kg, with a median of 0.03 mL/kg, a minimum of 0 mL, and a maximum of 2.94 mL/kg. These values were reported by the surgeon who operated. The subjective evaluation of the patient’s bleeding conducted by the anesthesiologist was always normal, while the surgeon identified three patients (2.9%) with bleeding higher than normal.

##### 3.2.1.4 Data describing temperature management

The passive temperature management techniques used included covering the patient with a cotton surgical drape (95%) or with an anti-decubitus pad (blanket) (65.7%). The active temperature management techniques used included the hyper-hypothermia water mattress system (Blanketrol) with an average setup temperature of 38.7°C (33.3%), warmed infusions (16.7%), a warm air heating system (7.8%), and an infusion tube heater (2%). The average operating room temperature was 25.1°C, with a median of 25.4°C and a standard deviation of 0.75°C (minimum 22.8°C, maximum 28°C). The temperature of the operating theatre was actively increased in one case. Prewarming was not performed in our patients.

Only passive methods of temperature management were used in 39% of the patients, while one active method was used in 36% of the patients, two active methods in 22%, and three active methods in 3%.

#### 3.2.2 Incidence of coagulation disorders

The incidence of abnormalities in the individual parameters of the coagulation tests ranged from 5.9 to 32.4%. The ROTEM results were abnormal in 18.7% of the cases (18.4% in the first measurement and 19% in the second), and the standard coagulation tests showed abnormalities in 15.9% of the cases (14.2% in the first measurement and 17.7% in the second). A more detailed description of the abnormalities in the individual parameters of the coagulation tests is shown in Table 4 (ROTEM and standard coagulation tests: Results within the normal range).

**Table 4:** ROTEM and standard coagulation tests: results within the normal range.

ROTEM and standard coagulation tests:			
Results within the normal range			
EXTEM CT 1	9.80%	FIBTEM A10 1	15.69%
EXTEM CT 2	5.88%	FIBTEM A10 2	16.67%
EXTEM CFT 1	31.37%	FIBTEM A20 1*	15.69%
EXTEM CFT 2	32.35%	FIBTEM A20 2*	26.47%
EXTEM α 1	30.39%	FIBTEM MCF 1	20.59%
EXTEM α 2	20.59%	FIBTEM MCF 2	24.51%
EXTEM A10 1	16.67%		
EXTEM A10 2	16.67%	aPTT-R 1	11.76%
EXTEM A20 1*	11.76%	aPTT-R 2	8.82%
EXTEM A20 2*	11.76%		
EXTEM MCF 1	13.73%	PT - R 1	16.67%
EXTEM MCF 2	15.69%	PT - R 2	26.47%

*Adult reference values.

**Figure 3:**
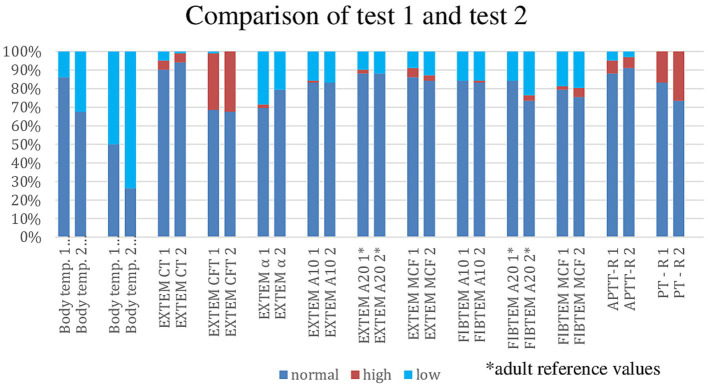
Comparison of TEST 1 and TEST 2: normal, decreased, and elevated values.

The values within and outside the normal range from the first and second measurements of body temperature at the time of sampling (using cutoff temperatures below 36.5°C and 36.0°C), as well as the results from the coagulation tests (ROTEM, APTT, and PT), are shown in Figure 3 (Comparison of TEST 1 and TEST 2: normal, decreased, and elevated values). Comparisons of the individual values from EXTEM and FIBTEM did not reveal statistically significant differences.

##### 3.2.2.1 Data analysis using statistical tests

##### 3.2.2.2 Difference between values from TEST 1 and TEST 2

In the Wilcoxon matched-pairs test, the PT-R values from measurements 1 and 2 were statistically different (*p* < 0.001), while the aPTT-R values from measurements 1 and 2 were marginally non-significant (*p* = 0.071). All other values were not statistically significant. To investigate the potential influence of body temperature variation on coagulation profiles, we assessed the relationship between changes in temperature and corresponding alterations in coagulation parameters. To ensure temporal concordance between physiological and laboratory data, the body temperature difference (ΔT) between the first and final blood sampling was calculated. A statistically significant negative correlation, based on Spearman’s correlation coefficient, was observed between the change in body temperature (ΔT) and changes in the following EXTEM parameters: ΔEXTEM A10 (*p* = 0.018), ΔEXTEM A20 (*p* = 0.013), and ΔEXTEM MCF (*p* = 0.002). No statistically significant correlations were found between ΔT and the following parameters: ΔEXTEM CT, ΔEXTEM CFT, ΔEXTEM *α*, ΔFIBTEM CT, ΔFIBTEM α, ΔFIBTEM A10, ΔFIBTEM A20, ΔFIBTEM MCF, ΔaPTT-R, and ΔPT-R. Subsequently, we determined the changes in coagulation parameters (e.g., ΔaPTT-R and ΔPT-R) over the same interval and performed a correlation analysis to evaluate their association with the observed temperature variation.

##### 3.2.2.3 Minimal body temperature during surgery, coagulation, and other variables

Pearson’s chi-squared test was used to assess the occurrence of abnormalities in the PT-R and aPTT-R in relation to Tmin thresholds of <36.5°C and <36°C. In both analyses, *p*-values exceeded 0.05, indicating no statistically significant association between these variables.

In a binary logistic regression analysis, the following parameters were included: age, operating theatre temperature, EXTEM 2 CT, EXTEM 2 A10, and FIBTEM 2 A 10 in relation to body temperature below 36.5°C (Tmin below 36.5°C). The results did not show that any of these variables were significantly affected by a body temperature below 36.5°C. The variable age had a *p*-value of 0.053. Spearman’s correlation coefficient confirmed these results; age was slightly significant, with a p-value of 0.015 (correlation was found to be significant at the 0.05 level (two-tailed)). The younger the children, the less likely they were to experience a lower body temperature.

Using a temperature threshold of Tmin below 36°C yielded very similar results. In this binary logistic regression analysis, only age emerged as a significant factor, with a *p*-value of 0.028.

The relationship between abnormalities in the EXTEM 2 and FIBTEM 2 tests, as well as age-related reference values combined with Tmin, was statistically insignificant, with *p*-values higher than 0.05 in all cases. The correlation between FIBTEM A20 and Tmin had a p-value of 0.067, indicating it was not statistically significant.

##### 3.2.2.4 Body temperature (TEST 2), coagulation, and other variables

Pearson’s chi-squared test was used to assess the occurrence of abnormalities in the PT-R and aPTT-R in relation to body temperature 2 thresholds below 36.5°C and 36°C. In both analyses, *p*-values exceeded 0.05, indicating no statistically significant association between these variables.

In the binary logistic regression analysis involving age, operating theatre temperature, EXTEM 2 CT, EXTEM 2 A10, and FIBTEM 2 A10 in relation to body temperature 2 below 36.5°C, all parameters were statistically insignificant, with *p*-values exceeding 0.05. The correlation between age and body temperature 2 had a *p*-value of 0.075. When using body temperature 2 below 36°C as a threshold, the correlation between operating theatre temperature and body temperature 2 was statistically significant, with a *p*-value of 0.037.

##### 3.2.2.5 Relationship between EXTEM-CT and PT-R

We tested the relationship between EXTEM-CT and PT-R using Pearson’s chi-squared test. The results for EXTEM-CT1 and PT-R1 did not match and were statistically non-significant, with a p-value of 0.29. Similarly, the results for EXTEM-CT2 and PT-R2 were also statistically non-significant.

##### 3.2.2.6 Percentage differences in the test results using age-dependent and age-independent limits

**Figure 4:**
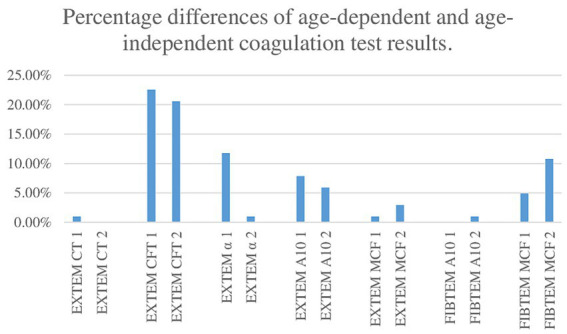
Percentage differences in age-dependent and age-independent coagulation test results.

The largest discrepancy between age-specific (14) and age-non-specific reference range evaluations was observed in the EXTEM CFT test. For further details, see Figure 4 (Percentage differences of age-dependent and age-independent coagulation test results).

## 4 Discussion

Our study investigated the relationship between perioperative hypothermia and coagulation status in pediatric patients undergoing arthroscopic and open abdominal surgeries. Our findings indicated that while mild hypothermia—defined as a body temperature below 36.0°C—did not manifest as clinically significant coagulation disorders requiring intervention, the dynamic nature of hemostasis presented challenges in accurately assessing coagulation status during the surgery. In our cohort, we observed a notable incidence of coagulation abnormalities assessed through standard tests and ROTEM, with results ranging from 5.9 to 32.4%. However, we did not identify statistically significant changes in coagulation parameters throughout the surgical procedure, except for the PT-R. Furthermore, our study aligns with existing literature suggesting that traditional coagulation tests, such as PT and aPTT, often fail to correlate well with more dynamic assessments, such as ROTEM, particularly in the context of mild hypothermia (15). Although we did not detect any clinically significant coagulation disorders during episodes of mild hypothermia, this does not necessarily indicate an absence of effects on coagulation. While the PT-R was the only parameter that showed a statistically significant change, this finding suggests that even mild hypothermia may have an impact on coagulation. This temperature reduction might be tolerable in the context of the surgeries we studied. However, the clinical relevance of this finding remains uncertain, particularly in more vulnerable populations, such as neonates or critically ill children. Future studies should focus on a broader range of coagulation markers to clarify this relationship. While our data suggest that effective perioperative temperature management may help mitigate the risk of coagulopathy, it remains unclear whether mild intraoperative hypothermia within the observed range has clinically relevant consequences. Therefore, our findings should be interpreted within the context of the study. One possible explanation for the lack of widespread coagulation changes is the short duration of hypothermia and the ability of the pediatric patients to maintain effective coagulation through compensatory mechanisms. In addition, it is possible that standard coagulation tests do not fully capture subtle functional changes that might occur under hypothermic conditions. Laboratory-based coagulation tests performed at standard laboratory temperature (typically 37°C) may not accurately reflect the hemostatic status of hypothermic patients (16–18). However, viscoelastic testing methods allow for temperature-adjusted measurements that better simulate *in vivo* conditions. In our study, we predefined a protocol for dual ROTEM testing—at the patient’s actual temperature and at 37°C—for cases where body temperature dropped below 34°C. However, since no such cases occurred, all tests were performed at 37°C, and a temperature-adjusted comparison was not necessary.

While this study provides valuable insights, a few limitations should be acknowledged. A number of participants (25%) were excluded due to incomplete data or technical issues with the ROTEM device. In addition, the predominance of orthopedic patients limits the diversity of surgical procedures represented, although it still captures key pediatric surgeries. While the study covered a broad age range (0–18 years), the younger age groups, particularly, had no or fewer patients, which might have reduced the statistical power and generalizability of the findings for these subgroups. The limited sample size in the younger age categories could make it difficult to draw definitive conclusions about the effects of surgical interventions and temperature regulation in the youngest patients. The absence of patients with hematological disorders or those on medications affecting coagulation limits the applicability of the results to certain clinical populations, but the study’s findings remain applicable to the general pediatric population. Variability in the temperature measurement methods could have introduced some inconsistency in the temperature data, although the majority of measurements were obtained using the more reliable nasopharyngeal method. The study tracked the use of temperature management tools during surgery, but it did not standardize these methods. This variability could have affected the body temperature outcomes. The study was conducted at a single center (University Hospital Brno), where unique factors such as operating room temperatures and anesthesia protocols could have affected the results. Another limitation of our study is that the analysis of shivering was performed only in the patients undergoing knee arthroscopy, while the patients with open abdominal surgery were excluded due to the small sample size, preventing statistical comparison. Although this subgroup was included in the overall study analysis, our findings on shivering cannot be directly applied to them. Similarly, in the analysis of coagulation, we focused on global hypothermic effects rather than regional temperature differences; therefore, we did not differentiate between patients with and without a tourniquet. This limits the external validity of the study, as the findings may not be applicable to hospitals with different environmental conditions or practices. While these factors introduce some limitations to the study’s generalizability, they do not critically undermine the overall findings and conclusions. Future studies with larger, more diverse samples and standardized methods would help to strengthen and expand upon these results.

An incidence of minimal body temperature below the normal threshold of 36.5°C during the surgery was observed in 84.3% of the cases. Hypothermia, defined as a body temperature below 36.0°C, occurred in 42.2% of the cases. These findings are consistent with previously published studies reporting an incidence of hypothermia between 45 and 85% (3–5), with the highest incidence observed in a cohort of neonates at 87.6% (5). In the first part of the study Peritemp performed at our department, which included all types of surgeries lasting more than 30 min, we found a body temperature below 36.5°C in 66% of pediatric patients and below 36.0°C in 36% (13). This confirms that hypothermia is a common phenomenon during the perioperative period.

The most frequently used method was the nasopharyngeal sensor (71% of the cases). It is technically well available during orthopedic, urologic, and general surgeries and is also the most commonly used method in European surveys (19). This technique allows measurement in well-perfused sites, reflecting core body temperature. Core temperature thermometers may have an acceptable error margin of ±0.5°C from the true core temperature (2). True core temperature is represented by measurements from the pulmonary artery, distal esophagus, nasopharynx, and tympanic membrane (2) or by using a zero-heat-flux thermometer placed on the forehead (20). Near-core temperature can be measured orally or axillary (21). Bladder and rectal temperatures are less accurate (2), and in our study, none of the anesthesiologists opted for these methods.

Core temperature during general anesthesia typically follows a predictable pattern. It can be described in three phases, as confirmed by Matsukawa et al. (22) in a study involving six volunteers undergoing general anesthesia. Phase I is characterized by a rapid decrease in temperature during the first hour of anesthesia, probably caused by heat redistribution. This is followed by Phase II, a slower, linear decline in temperature, during which heat loss primarily occurs through radiation and convection, compounded by a reduced metabolic rate. Phase III, the plateau phase, typically begins after 3 to 4 h, when further heat loss is prevented by vasoconstriction (22). Additional decreases in temperature can occur after another redistribution of heat, such as after tourniquet release (23). Sessler et al. (7) stated that redistribution has relatively little effect in pediatric patients, as infants and small children have such small extremities that most of their bodies can be considered core. In our study, the mean and median body temperature values showed a gradual decrease, with a rapid decline occurring during the first hour of anesthesia. The plateau phase did not occur due to the relatively short duration of anesthesia. Temperatures remained within the normal and mild hypothermia ranges, with typical reductions of 1°C to 3°C (24). We observed a 26.4% incidence of postoperative shivering, which falls within the 5 to 65% range reported in the literature (25). Duration of surgery was identified as an independent risk factor for postoperative shivering. Similarly, a study conducted by Eberhart et al., (26) which included 1,340 patients, confirmed a significant association between prolonged surgical time and an increased incidence of postoperative shivering. Shirozu et al. (27) examined patients with a core temperature ≥ 36.5°C and found that longer surgeries increased the risk of shivering. They also reported that shivering was linked to peripheral temperature rather than core temperature, with fingertip temperature being lower in the shivering group (34.4°C vs. 35.4°C).

Blood loss varied among the patients of different ages and with different blood volumes, undergoing various surgical procedures performed by different surgeons, making it unfeasible to assess the correlation between blood loss, hypothermia, and mild coagulopathy.

Surgery significantly impacts patient coagulation systems due to various factors. Tissue injury (28), blood loss, hemodilution (29), and other surgery factors lead to a tendency toward bleeding or thrombosis (29). Perioperative factors such as hypothermia (30), metabolic acidosis (31), and extracorporeal circulation (29) also interfere with coagulation. Patient-related factors such as cancer (32), infections (10), medications (anticoagulants, antiplatelet, or fibrinolytic drugs, NSAIDs, SSRIs, and ATBs), and coexisting bleeding disorders (von Willebrand disease, thrombocytopenia, etc.) can aggravate coagulopathy. Such perturbations in coagulation can be assessed using various laboratory assays.

We evaluated coagulation disorders during surgery using both standard tests and ROTEM, with individual parameter abnormalities ranging from 5.9 to 32.4%. Preoperative abnormal ROTEM results have been previously evaluated by Ghavidel et al. in a cohort of adult patients undergoing coronary artery bypass grafting (CABG). In their study, 2.8% of patients had abnormal preoperative ROTEM results, compared to 33.3% in the group with abnormal postoperative bleeding. However, the authors did not specify which ROTEM parameter values fell outside the reference range (33). In our cohort, we did not identify statistically significant changes in coagulation parameters at the beginning and before the end of anesthesia, except for the PT-R. While the PT-R demonstrated significant changes, EXTEM did not show statistical significance, although both tests theoretically assessed similar aspects of coagulation (34). A potential explanation for this discrepancy could be the influence of fibrinogen concentration and platelet function on EXTEM (35). Cho et al. (36) conducted a study on 106 pediatric patients (aged 3 months to 17 years) undergoing liver transplantation and found that an EXTEM MCF of 30 mm predicted a platelet count <30,000/mm3 and a FIBTEM value of 6 mm predicted a fibrinogen concentration <100 mg/dL. aPTT and PT were significant but only weakly correlated with clotting time on ROTEM®. Haas et al. found that standard coagulation tests such as aPTT and PT often do not correlate well with ROTEM results, except for fibrinogen levels assessed with FIBTEM. Early abnormalities in aPTT or PT, commonly seen during intraoperative bleeding and hemodilution, may not always indicate significant bleeding (15). This aligns with our findings of pathologically elevated coagulation test results without clinical manifestations. To further explore the impact of temperature on coagulation dynamics, we evaluated correlations between perioperative body temperature changes and changes in coagulation parameters. A statistically significant negative correlation was observed between the changes in body temperature and ΔEXTEM A10, ΔEXTEM A20, and ΔEXTEM MCF. These parameters reflect clot firmness and are likely influenced primarily by platelet-related mechanisms rather than fibrin polymerization. This interpretation is supported by the absence of significant correlations for FIBTEM, as well as for initiation and propagation parameters, such as EXTEM CT, CFT, and *α*-angle, and for conventional coagulation assays. These findings support the hypothesis that temperature variation may primarily influence the later phases of clot formation. These findings could be attributed to the dynamic nature of hemostasis, where both bleeding and clot formation represent the extremes with many subtle variations in between. The levels of these mediators are known to fluctuate rapidly, and their degree of perturbation is dependent not only on the type and duration of surgery but also on the timing of blood collection (29). Larsen et al. (37) in a systematic review, found that laboratory coagulation tests have limited value in predicting intra- or postoperative bleeding, although whole blood coagulation tests are important in managing bleeding surgical patients.

Our study confirmed the previously established correlation between operating room temperature and body temperature below 36°C at the end of surgery. This also corresponds to the correlation observed in our first study (13). Operating room (OR) temperature is one of the main factors contributing to the decrease in core temperature in neonates and infants (38). The median OR temperature was 25.0°C, with a minimum of 22.8°C and a maximum of 28.0°C. In a study by Tander et al., the central temperature of infants and newborns during anesthesia decreased significantly when the room temperature was below 23°C. In neonates, core temperatures are less stable regardless of OR temperature and type of surgery. At high OR temperatures, infants can stabilize their core temperature better than neonates (38). In a study by Ozer et al. (39) on adult patients undergoing general anesthesia at room temperatures of 21–22°C and 23–25°C, there was no significant difference in tympanic temperatures across all methods of anesthesia. In our group of pediatric patients, the median age was 16 years, with a minimum age of 1.5 years. This suggests that the influence of operating room temperature is noticeable, indicating that not only newborns and infants but also older pediatric patients are susceptible to a decrease in body temperature.

A significant risk factor for a drop in body temperature or hypothermia during the surgery, defined as a minimum body temperature below 36.5 or 36°C, was higher age among the pediatric patients. One possible explanation is better temperature management in these patients. Furthermore, while pediatric patients are generally more susceptible to temperature fluctuations, as shown in both Tander et al.’s (38) and our findings, temperature management strategies can effectively reduce the incidence of inadvertent hypothermia in all age groups. For example, studies in pediatric populations have demonstrated a reduction in hypothermia incidence from 48 to 20% (40) and from 24 to 2% (41), following the implementation of temperature management protocols. This supports the conclusion by Neméth et al. (6) that the incidence of hypothermia is more strongly influenced by the warming strategy than by factors such as age or type of surgery.

When comparing age-dependent and age-independent reference ranges, we found observed discrepancies in the results, especially for EXTEM CFT. The authors of the age-related reference ranges, Oswald et al., investigated a cohort of 359 healthy children and found that extrinsically activated clot firmness was similar across all age groups (14). Although EXTEM CFT values fall within the reference ranges for children, it is evident that results can vary across specific age groups. This suggests that age-specific reference ranges should be utilized to ensure more accurate interpretation and diagnosis in pediatric patients.

In summary, our study focused on the relationship between perioperative temperature management and coagulation. While mild hypothermia was common, it did not result in widespread coagulation abnormalities, apart from a significant change in the PT-R.

## Data Availability

The raw data supporting the conclusions of this article will be made available by the authors, without undue reservation.
